# Predictors of response to erenumab after 12 months of treatment

**DOI:** 10.1002/brb3.2260

**Published:** 2021-07-16

**Authors:** Carlo Baraldi, Flavia Lo Castro, Maria Michela Cainazzo, Luca Pani, Simona Guerzoni

**Affiliations:** ^1^ PhD School in Neuroscience Department of Biomedical Metabolic and Neural Sciences University of Modena and Reggio Emilia Modena Italy; ^2^ Post‐graduate School of Pharmacology and Clinical Toxicology Department of Biomedical Metabolic and Neural Sciences University of Modena and Reggio Emilia Modena Italy; ^3^ Medical Toxicology‐headache and Drug Abuse Research Center Department of Biomedical Metabolic and Neural Sciences University of Modena and Reggio Emilia Modena Italy; ^4^ Pharmacology Unit Department of Biomedical Metabolic and Neural Sciences University of Modena and Reggio Emilia Modena Italy; ^5^ Department of Psychiatry and Behavioral Sciences University of Miami Miami Florida USA; ^6^ VeraSci Durham North Carolina USA

**Keywords:** chronic migraine, erenumab, medication overuse‐headache, predictors of response, real‐life setting

## Abstract

**Objective:**

Erenumab is a monoclonal antibody acting against calcitonin gene‐related peptide receptor and approved for the preventive treatment of chronic migraine. The aim of the present study is to identify clinical predictors of good response in patients with chronic migraine and medication overuse‐headache.

**Material and methods:**

This was a retrospective single‐center not funded study. Enrolled patients were affected by chronic migraine and medication overuse‐headache treated with erenumab monthly, up to 1 year. At 1 year, patients were classified as good responders if they displayed a ≥50% reduction in the number of headache days per months compared to the baseline.

**Results:**

After 1 year, a significant improvement in the number of headache days per months, analgesic consumption, 6‐items headache impact test, and migraine disability assessment questionnaire scores were obtained compared to the baseline. Patients who obtained a ≥50% reduction in the number of headache days per month compared to the baseline displayed a longer history of medication overuse‐headache, a higher number of painkillers taken per month at the baseline and a higher number of failed preventive treatments in the past.

**Conclusions:**

Patients with longer medication overuse‐headache duration, higher analgesic intake, and a higher number of previous preventive treatment failures may receive less benefit with erenumab.

## Graphical abstract

Patients affected by chronic migraine and medication overuse headache and treated with Erenumab for one year were more likely to be ≥50% responders if they had a longer duration of medication overuse‐headache, a higher number of painkillers taken per month and if had failed a higher number of preventive treatments for migraine.

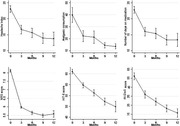

## 1 INTRODUCTION

According to the International Classification of Headache Disorders, 3rd Edition (ICHD‐3), chronic migraine (CM) is characterized by the recurrence of ≥15 headache days per month, of which ≥8 days with migraine features, for at least 3 months (Headache Classification Committee of the International Headache Society (IHS) 2018). CM sufferers often overuse painkillers to treat frequent migraine attacks, thus worsening CM itself and generating a secondary headache called medication overuse‐headache (MOH) (Diener et al., 2016). CM complicated with MOH affects about the 1%–2% of the general population and imposes a significant burden on the society (Lanteri‐Minet et al., 2011). Moreover, the management of this condition is difficult and based, usually, on a bimodal approach: a painkiller withdrawal to stop medication overuse and the prescription of preventive treatment for CM (Carlsen et al., 2018). According to the European Headache Federation (EHF), topiramate, onabotulinumtoxinA (BT‐A), and monoclonal antibodies targeting calcitonin gene‐related peptide (CGRP) or its receptor are approved for the preventive treatment of CM (Steiner et al., 2019). Among these, erenumab has demonstrated good efficacy and a favorable safety profile in a sub‐group analysis of CM and MOH sufferers from a randomized placebo‐controlled trial (Tepper et al., 2019). Moreover, erenumab was effective and safe in treating patients with CM complicated with MOH in a real‐life setting, up to 1 year (Cainazzo et al., 2021). The continuous long‐term use of erenumab should be performed in order to avoid the relapse of CM and MOH, as preliminary findings seemed to suggest (De Matteis et al., 2021). Nevertheless, the long‐term use of erenumab raises some issues, such as its expensiveness, that may limit its affordability. Additionally, the long‐term safety of erenumab among CM sufferers was primarily explored in randomized controlled trials (RCTs) (Tepper et al., 2020) with restrictive inclusion criteria, thus risking to not “mirror” the population treated in real‐life settings (Heneghan et al., 2017). Indeed, in real‐life settings, severe constipation, asthenia, and vertigo were more common than in RCTs, thus imposing the careful evaluation of continuing treatment, especially in the case of a poor response (Kanaan et al., 2020). Due to this, it would be useful to define clinical features associated with a good response to erenumab in a so difficult‐to‐treat population, such as CM and MOH sufferers. Other groups have already explored response predictors to erenumab, but not on CM and MOH sufferers and for limited periods of time (Barbanti et al., 2020). We decided to perform an ancillary analysis on data collected for another study exploring the effectiveness and safety of erenumab in a real‐life setting, as to identify the clinical predictors of good response to erenumab after 1 year of therapy in patients affected with CM and MOH.

## 2 MATERIALS AND METHODS

### 2.1 Patients

This was a retrospective, not‐funded, single‐center study, performed at the Medical Toxicology‐Headache and Drug Abuse Research Centre of the University of Modena and Reggio Emilia. Patients affected by CM complicated with MOH who received erenumab for the preventive treatment of CM for 1 year between April 20, 2019 and July 31, 2020 were considered for enrollment. Treated patients were aged between 18 and 65 years and had failed or were not eligible to, at least, three classes of first‐choice preventive treatments for migraine, according to the European guidelines (amitriptyline, flunarizine, beta‐blockers, anticonvulsants, and BT‐A) (Steiner et al., 2019). Patients were enrolled after July 31, 2020, during a scheduled visit to the center, when they also signed an informed consent for study participation and data publication. Patients’ data were obtained by their electronic medical records, stored at the center. This study was approved by the Area Vasta Emilia Nord ethics committee (protocol number: 50/2020/OSS/AOUMO). All procedures were carried out following the latest version of the Declaration of Helsinki. This study is a secondary analysis of another one exploring the effectiveness and safety of erenumab in the treatment of CM and MOH in a real‐life setting.

### 2.2 Procedures

Erenumab was administered monthly at an initial dose of 70 mg and eventually titrated up to 140 mg from the fourth injection onwards, in the case the patient displayed a <30% reduction in the number of headache days (NHD) during the first 3 months of treatment, as a previous work suggested (Sacco et al., 2019). Patients were treated with erenumab up to 1 year, unless they decided to abandon it due to poor effectiveness, scarce tolerance, or well‐being. The development of an adverse event (AE) of, at least, moderate gravity, caused treatment discontinuation. During the treatment period, patients underwent a maximum of five visits, unless they withdrew due to one of the above‐mentioned reasons. At the baseline visit, patients’ sex, age, age of migraine onset, age of migraine chronification, duration for medication overuse, the presence of migraine with aura, the type of painkillers used, the number and type of previously failed preventive treatments, and the reasons (ineffectiveness or AEs) as well as comorbidities were collected. At the first visit and during the following ones, other variables were collected: the number of headache days per month in the last 3 months (NHD), the average number of painkillers taken per month in the last 3 months (analgesic consumption‐AC), the average number of days per month in which the patient took, at least, one painkiller, referring to the previous 3 months (number of days on medication [NDM]), and the average intensity of headache using the numeric rating scale score in the previous 3 months (NRS). Additionally, patients were asked to fill‐in the 6‐items headache impact test (HIT‐6) and the migraine disability assessment (MIDAS) questionnaire at every visit. Patients were continuously monitored for the development of AEs.

### 2.3 Statistical analysis

Continuous variables were expressed as mean ± standard deviation (SD), while categorical ones as subject counts and percentages. Continuous variables were tested for normality using the Shapiro–Wilk test. Normally distributed variables were compared with the one‐way analysis of variance followed by the Tukey–Kramer post hoc comparison test, otherwise a Kruskal–Wallis rank‐signed test was used. For multiple comparisons, the Bonferroni's correction was applied. Categorical variables were compared using the *χ* ^2^ test for the homogeneity of odds. Patients were considered good‐responders if they displayed a ≥50% reduction in the NHD, compared to the baseline. This cut‐off was chosen because it had been accepted as a threshold for good responders in other studies on erenumab (Raffaelli et al., 2020). Patients who discontinued erenumab due to ineffectiveness or AEs before reaching the year of treatment were considered as poor‐responders. Baseline characteristics were compared between good‐responders and poor‐responders at 12 months of treatment. A multiple logistic regression with backward elimination was then performed with all variables significantly associated with good responders’ rate at the univariate analysis. The model was tested for collinearity using the phi correlation coefficient, and collinear variables were eliminated from the model. Additionally, the Pearson's *χ* ^2^ goodness of fit test was carried out in order to assess the goodness of fit of the entire model. Additionally, the receiver operating characteristic curve (ROC) analysis was performed upon the entire model. Sample size was not calculated since this study was based on available data. *p*‐Values < .05 were considered significant. Statistical analysis was performed with STATA Ic15 software.

## 3 RESULTS

### 3.1 Demographic data

Data from 111 patients was elaborated, all of whom were affected by CM complicated with MOH, according to the international guidelines (Headache Classification Committee of the International Headache Society [IHS], 2018). The analyzed sample showed an average age of 50.58 ± 8.74 years and was composed mainly by females, about half of the latter were in menopause. Migraine lasted for a mean time of 33.58 ± 11.52, while the average CM duration was 14.53 ± 10.78. MOH lasted for an average time of 8.79 ± 8.05 years, and the majority of patients were triptan overusers (87.39%). The average number of failed preventive treatments was high (6.3 ± 2.21). Eighty‐three patients failed topiramate (74.77%) and 39 failed BT‐A (35.14%). Erenumab was given together with other preventive treatments in more than a half of the patients. The 18.02% of the sample underwent a painkiller withdrawal before starting erenumab. Ninety‐nine patients suffered from, at least, one comorbidity (89.19%), of whom 40 suffered from depression (36.04%), 35 from anxiety (31.53%), and 13 from fibromyalgia (11.71%). At the baseline, patients displayed an almost daily headache and took even more than one painkiller per day. The average NRS score at the beginning was 8.07 ± 1.52, while the mean values for the HIT‐6 and the MIDAS scores indicated a severe impairment of the patients’ quality of life. Erenumab was titrated up to 140 mg in 51 patients (49.51%), equally distributed between good‐responders and poor‐responders (OR = 0.51; 0.22 ÷ 1.19, *p* = .1125). Baseline data are summarized in Table 1.

**TABLE 1:** Baseline characteristics

Variable	Value
Number of patients	111 (100%)
Age	50.58 ± 8.74
Females	88/111 (79.28%)
Menopause	45/88 (51.14%)
Age of menopause	48.57 ± 3.42
Aura	28/111 (25.23%)
Allodynia	27/111 (24.11%)
Migraine duration	33.58 ± 11.52
CM duration	14.53 ± 10.78
Medication overuse duration	8.79 ± 8.05
Overused analgesics	
Triptans	97/111 (87.39%)
NSAIDs	44/111 (39.64%)
Combinations	13/111 (11.71%)
Number of preventive treatment failed	6.3 ± 2.21
Failed topiramate	83/111 (74.77%)
Failed BT‐A	39/111 (35.14%)
Comorbidities	99/111 (89.19%)
Anxiety	35/111 (31.53%)
Depression	40/111 (36.04%)
Fibromyalgia	13/111 (11.71%)
Erenumab in add‐on	61/111 (54.89%)
Detoxification	20/111 (18.02%)
NHD	23.16 ± 6.69
AC	40.41 ± 34.76
NDM	23.08 ± 6.88
NRS	8.16 ± 0.87
HIT‐6	65.5 ± 6.63
MIDAS	69.49 ± 20.08

Abbreviations: AC, analgesic consumption; BT‐A, onabotulinumtoxinA; CM, chronic migraine; HIT‐6, 6‐items headache impact test; NDM, number of days on medication; NHD, number of headache days; NRS, numeric rating scale; NSAIDs, nonsteroidal anti‐inflammatory drugs; MIDAS, migraine disability assessment.

### 3.2 Changes in NHD, AC, NDM, HIT‐6, MIDAS scores, and registered AEs

**FIGURE 1:**
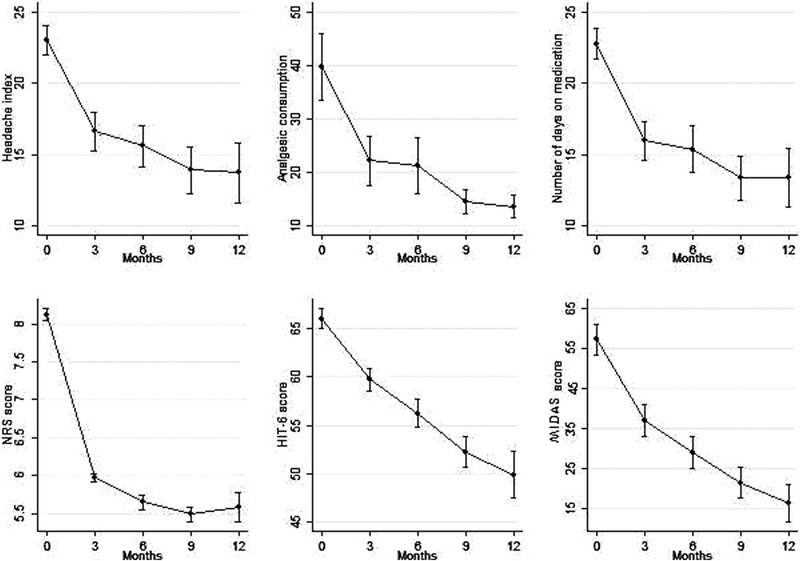
Number of headache days (NHD), analgesic consumption (AC), number of days on medication (NDM), 6‐items headache impact test (HIT‐6)‐score, and migraine disability assessment (MIDAS)‐score at every time‐point

At the 12th month, the NHD (23.16 ± 6.69 vs. 11.57 ± 8.94, *p* = .0001), AC (40.41 ± 34.76 vs. 11.51 ± 9.28, *p* = .0001), NDM 5(23.08 ± 6.88 vs. 11.35 ± 8.85, *p* = .0001), HIT‐6 score (65.5 ± 6.63 vs. 52.81 ± 9.76, *p* = .0001), and MIDAS score (69.49 ± 20.08 vs. 17.36 ± 13.42, *p *= .0001) were all significantly lower than the baseline. These data are summarized in Figure 1. AEs were mostly mild, with constipation as the most frequent, while four patients dropped out of treatment due to AE (two with moderate lower back pain, one with thoracic pain, and one with vertigo). All the AEs that caused treatment suspension subsided after erenumab withdrawal. These data are summarized in Table 2.

**TABLE 2:** Systemic adverse events

Adverse event	Number
Abdominal pain	1/111 (0.9%)
Athenia	4/111 (3.6%)
Constipation	32/111 (28.83%)
Disgeusia	2/111 (1.8%)
Flu‐like symptoms	3/111 (2.7%)
Laringodinia	1/111 (0.9%)
Low back pain	2/111 (1.8%)
Muscular pain	1/111 (0.9%)
Nausea	5/111 (0.5%)
Thoracic pain	1/111 (0.9%)
Vertigo	1/111 (0.9%)
Total	49/111 (44.14%)

### 3.3 Comparison between <50% responders and ≥50% responders

Considering the NHD, 49 patients were classified as <50% responders (49/111, 44.14%). The proportion of females was higher among the <50% responders (45/49 vs 43/62, *p* = .0039). Additionally, the duration of medication overuse was significantly higher among the <50% responders (234.49 ± 483.63 vs. 53.34 ± 48.39, *p* = .0001). The number of previously failed preventive treatments was significantly higher among <50% responders (7.86 ± 1.85 vs. 5.06 ± 1.62, *p* = .0001). NHD (25.39 ± 6.74 vs. 21.4 ± 6.15, *p* = .0018), AC (58.67 ± 44.55 vs. 26.13 ± 12.12, *p* = .0001), NDM (25.45 ± 6.77 vs. 21.21 ± 6.42, *p* = .0011), and MIDAS score (76.16 ± 20.23 vs. 64.21 ± 18.46, *p* = .0012) were significantly higher among <50% responders at the baseline. All these data are summarized in Table 3. At the multivariate logistic analysis, only the MOH duration, the number of previous preventive treatments failed, and the AC at the baseline remained significantly higher in <50% responders than in ≥50% responders, controlling for other significant variables at the univariate analysis. In particular, the odds of being a poor responder increased by 0.24 [0.12 ÷ 0.37] (*p* < .0001) for every year of increase in MOH duration. The odds of being a <50% responder increased by 1.04 [0.06 ÷ 2.01] (*p* = .042) for every 15 analgesic taken. Additionally, the odds of being a <50% responder increased by 0.87 [0.41 ÷ 1.34] (*p* < .0001) for every preventive treatment in which the patient had failed in the past. The Pearson *χ* ^2^ goodness of fit test gave a *χ* ^2^ value of 69.18 (*p* = .9947), suggesting a good fit of the model. The ROC curve analysis indicated that the whole multivariate logistic regression model had an area under the curve of 0.942, thus suggesting a good model predictivity of the responder status.

**TABLE 3:** Comparison of migraine‐related factors between responders and nonresponders at 12 months

Variable	Poor‐responders	Good‐responders	*p*‐value	OR	*p*‐value
Number	49/111 (44.14%)	62/111 (55.86%)	–	**–**	**–**
Age	50.84 ± 8.85	50.37 ± 8.72	.7733	**–**	**–**
Sex	45/49 (91.84%)	43/62 (69.25%)	**.0039**	0.38 [0.07 ÷ 2.12]	.275
Menopause	23/44 (52.27%)	22/44 (50%)	.8321	**–**	**–**
Age of menopause	48.41 ± 3.54	48.73 ± 3.37	.9299	**–**	**–**
Aura	12/49 (24.49%)	16/62 (25.81%)	.8745	**–**	**–**
Allodynia	12/49 (24.49%)	15/62 (24.19%)	.9713	**–**	**–**
Migraine duration	34.22 ± 10.39	33.06 ± 12.74	.6221	**–**	**–**
CM duration	16.47 ± 11.55	12.9 ± 9.88	.1158	**–**	**–**
Medication overuse duration	170.41 ± 102.44	53.34 ± 48.39	**.0001**	1.45 [1.09 ÷ 2.12]	**<.0001**
Number of preventive treatment failed	7.86 ± 1.85	5.06 ± 1.62	**.0001**	1.51 [1.06 ÷ 2.15]	**.021**
Failed topiramate	36/49 (73.47 %)	47/62 (75.81%)	.7793		
Failed BT‐A	20/49 (40.82%)	19/62 (30.65%)	.2672	**–**	**–**
Depression	13/49 (26.53%)	27/62 (43.55%)	.0649	**–**	**–**
Anxiety	16/49 (32.65%)	19/62 (30.65%)	.8219	**–**	**–**
Fibromyalgia	6/49 (12.24%)	7/62 (11.29%)	0.8771	**–**	**–**
Other comorbidities	46/49 (93.88%)	56/62 (90.32%)	.7623	**–**	**–**
Erenumab in add‐on	28/49 (57.14%)	33/62 (53.23%)	.6818	**–**	**–**
Detoxification	8/49 (16.33%)	12/62 (19.35%)	.6815	**–**	**–**
NHD	25.39 ± 6.74	21.4 ± 6.15	**.0018**	1.38 [0.39 ÷ 4.84]	.613
AC	58.47 ± 44.55	26.13 ± 12.12	**.0001**	0.93 [0.88 ÷ 0.99]	**.021**
NDM	25.45 ± 6.77	21.21 ± 6.42	**.0011**	0.77 [0.22 ÷ 2.67]	.681
NRS	8.22 ± 0.8	8.11 ± 0.93	.7105		
HIT‐6	65.08 ± 6.86	66.04 ± 6.35	.4816	**–**	**–**
MIDAS	76.16 ± 20.23	64.21 ± 18.46	**.0018**	1.11 [0.73 ÷ 1.69]	.613

Abbreviations: AC, analgesic consumption; BT‐A, onabotulinumtoxinA; CM, chronic migraine; HIT‐6, 6‐items headache impact test; NDM, number of days on medication; NHD, number of headache days; NRS, numeric rating scale; NSAIDs, nonsteroidal anti‐inflammatory drugs; MIDAS, migraine disability assessment.

The bold values are the significant P‐values, that is the P‐values lower than 0.05.

## 4 DISCUSSION

### 4.1 Effectiveness and safety

This study aims to explore clinical predictors of ≥50% response to erenumab after 12 months treatment. Beside this, the present study confirms the effectiveness and safety of erenumab, even in a severely impaired population at the baseline, mirroring the results obtained by other groups (Ornello, Casalena, Frattale, Gabriele, et al., 2020; Russo et al., 2020) (Figure 1). After 1 year, NHD, AC, NDM, HIT‐6, and MIDAS scores improved significantly compared to the baseline, confirming the effectiveness of erenumab in this severely impaired population. Furthermore, erenumab also displayed a good AEs profile since the majority of AEs were mild (Table 2).

### 4.2 Comparison between ≥50% responders and <50% responders

Enrolled patients displayed a long history of migraine, CM and MOH at the baseline. Moreover, the enrolled patients had almost daily migraine attacks, taking more than one painkiller per day and had failed many preventive treatments before starting erenumab (Table 1). Stating this, the high clinical impairment of the enrolled patients justifies the slightly lower percentage of ≥50% responders after one year than in other studies (55.86%) (Russo et al., 2020). Despite the general improvement, ≥50% responders were 62 (55.86%) and showed a higher number of females as well as a lower duration of medication overuse. Additionally, ≥50% responders had failed a lower number of preventive treatments in the past. Furthermore, the NHD, AC, NDM, and MIDAS scores were significantly higher among <50% responders (Table 3). These results agree partially with the ones achieved by Ornello and co‐workers: exploring the factors associated with a status of anytime responders in patients receiving erenumab, they found that responders showed a lower average number of headache days per months and a lower AC (Ornello, Casalena, Frattale, Gabriele, et al., 2020). Despite this, a successive study from the same group failed to demonstrate significant baseline differences between patients with CM who had remitted to episodic migraine after 6 months of treatment with erenumab and the ones who hadn't (Ornello, Casalena, Frattale, Cponnetto, et al., 2020). Regarding the other approved CM preventive treatments, Pozo‐Rosich's group discovered that the presence of MOH was significantly associated with a poor response to BT‐A, as well as the number of migraine days per month, migraine duration, the presence of aura, and the presence of anxiety (Alpuente et al., 2020; Dominguez, Pozo‐Rosich, Torres‐Ferrus, et al., 2018; Dominguez, Pozo‐Rosich, Leira, et al., 2018 ). Schiano di Cola et al. (2019) reached comparable results, assessing that depressive symptoms and the presence of MOH negatively predict the response to BT‐A. On the other hand, the presence of chronic daily headache, a history of chronic daily headache longer than 6 months, and a negative response to divalproex sodium predict a poor response to topiramate (Rothrock et al., 2005). Taking the above‐mentioned data together a higher number of migraine days per months, the presence of MOH and anxious and/or depressive symptoms seem to lower the effectiveness of BT‐A and topiramate. In this study, at the univariate analysis, no differences were found regarding CM duration between <50% and ≥50% responders, as well as in anxiety and/or depression prevalence among the two groups. Instead, a significantly longer history of MOH was detected among <50% responders, and this difference remained significant even at the multivariate analysis (Table 2). Medication overuse increases trigeminal excitability through the alteration of central serotonin and endocannabinoids descending modulating pathways (Srikiatkhachorn et al., 2014). On the other hand, data from pre‐clinical studies demonstrated that both triptans and non‐steroidal anti‐inflammatory drugs may enhance CGRP expression in the trigeminal ganglion in a rat model of MOH (Buonvicino et al., 2018). It may be reasonable that a long history of MOH in humans could increase CGRP levels, thus affecting the effectiveness of erenumab. It has been proven that the plasma levels of CGRP are higher among MOH sufferers and that detoxification restores them (Greco et al., 2020). Considering this, in MOH patients, the higher levels of CGRP and the blocking of CGRP receptor may force the available CGRP to bind to other receptors such as the amylin receptor or the vasoactive intestinal peptide one (Russell et al., 2014). This may sustain, at least in part, CGRP action, thus explaining the longer MOH duration of <50% responders observed in this study. A higher number of preventive treatment failures was found in poor responders in this study, and the significance has been maintained even at the multivariate analysis. It ought to be considered that patients with multiple preventive treatment failures represent a more refractory population: Ornello and co‐workers suggested that, due to the lower effectiveness of erenumab in patients who had failed previous preventive treatments, erenumab itself should be soon increased to 140 mg or even started at this dose (Kanaan et al., 2020). Moreover, to witness the higher refractoriness of patients who had failed many preventive treatments in the past, the LIBERTY trial was conducted with the 140 mg dose (Reuter et al., 2018).

Having failed preventive treatments in the past could be identified as a resistant sub‐group of patients. The latter could find less benefit with erenumab. Additionally, a higher NHD, AC, and NDM were found to be associated with a <50% response to erenumab, but only the AC resulted significantly at the multivariate analysis. This result is in line with the ones achieved by Ornello and co‐workers, who found a lower number of monthly migraine days and a lower NDM in erenumab responders after 6 months of treatment, compared with poor‐responders (Ornello, Casalena, Frattale, Gabriele, Cponnetto, et al., 2020). However, higher NHD and AC are associated with poor response toward other preventive treatments for CM, such as BT‐A (Dominguez et al., 2018) and topiramate (Srikiatkhachorn et al., 2014). Indeed, painkiller overuse lowers trigeminal activation threshold, so patients are at higher risk of developing migraine attacks (Buonvicino et al., 2018). Moreover, the repetitive activation of the trigeminal nerve may enhance the release of glutamate and CGRP even in the trigeminal nucleus caudalis, leading to central sensitization that is present in MOH sufferers (Perrotta et al., 2010). To date, no studies, to our knowledge, have linked the number of painkillers with the trigeminal sensitization, as these results suggest. The ROC curve analysis of the multivariate logistic regression model revealed an area under the curve of 0.942, thus suggesting a good predictivity of the responder status. Since this, the AC, MOH duration, and the number of preventive treatments failed were independently and significantly associated with ≥50% responder status, even considering the high overall goodness of fit of the entire model.

### 4.3 Limits of the study

The present study has some limits, such as its retrospective nature, which made the analyzed data unbalanced; for example, patients with a MOH history longer than 10 years were enrolled alongside patients with a shorter MOH history. The same issue may be present for AC and the total number of previous preventive treatments failed. Another limit of this study is the small sample size, which was not based on any calculation, due to the above‐mentioned retrospective nature of the study. The 30% reduction in NHD was chosen as a cut‐off to increase the dosage of erenumab to rule out that the natural fluctuation of CM could affect patients’ response (Serrano et al., 2017). Furthermore, another matter of debate may concern the choice of the ≥50% threshold to distinguish poor‐responders and good‐responders, even because a ≥30% threshold may also be used in CM. The ≥50% threshold was chosen because it is considered a clinical meaningful response (Silberstein et al., 2008) and to deeply characterize patients’ response, avoiding that the natural fluctuations of CM may affect the responder status (Serrano et al., 2017). It would have been useful to explore even the number of patients achieving a ≥75% reduction of the NHD compared to baseline, that is, excellent responders, but their number was low (20/111, 18.02%), not permitting a reliable comparison between patients who had achieved this level of response and the ones who had not. It should be considered that the 30% and the 50% threshold are very close, so the risk that Erenumab titration may have affected the results is possible. Anyhow, no differences were noticed between patients who switched to Erenumab 140 mg between good‐responders and poor‐responders.

## 5 CONCLUSIONS

In conclusion, the present study is the first one specifically exploring patients’ features associated with a ≥50% response to erenumab after 1 year of treatment and suggests that AC, MOH duration, and the number of previous preventive treatments failed may identify a subgroup of patients who may found less benefits with erenumab in long‐term treatments. So, in these patients the continuation of the treatment with erenumab should be critically evaluated after the first injections and the escalation to the 140 mg dose, especially in the case of scarce tolerability. Obviously, further, bigger studies are needed to deeply phenotype these patients, thus identifying clinical predictors of good and poor response to erenumab and other anti‐CGRP monoclonal antibodies.

## CONFLICT OF INTEREST

Carlo Baraldi received travel grants from Allergan, Teva, Novartis, and Ely Lilly. Simona Guerzoni received travel grants and honoraria from Allergan, Teva, Novartis, and Eli Lilly. Luca Pani is the Chief Scientific Officer of EDRA‐LSWR Publishing Company and of Inpeco SA Total Lab Automation Company. In the previous years, he served as a scientific consultant to AbbVie, USA; BCG, Switzerland; Boehringer‐Ingelheim, Germany; Compass Pathways, UK; Johnson & Johnson, USA; Takeda, USA; VeraSci, USA; and Vifor, Switzerland. Flavia Lo Castro and Maria Michela Cainazzo have no conflict of interest to declare.

### PEER REVIEW

The peer review history for this article is available at https://publons.com/publon/10.1002/brb3.2260.

## Data Availability

Data will be available from the corresponding author on reasonable request.
